# Cumulative Incidence of Methicillin-Resistant Staphylococcus aureus Colonization in Resident Physicians Over Time

**DOI:** 10.7759/cureus.17958

**Published:** 2021-09-14

**Authors:** Scott Gutovitz, Leslie Hart, Nayda Parisio-Poldiak, Morgan Smith, Lexus Dickson, Steven Warrington, Stephen Thacker, Amanda Janke

**Affiliations:** 1 Emergency Medicine, Grand Strand Medical Center, Myrtle Beach, USA; 2 Department of Health and Human Performance, College of Charleston, Charleston, USA; 3 Graduate Medical Education, Grand Strand Medical Center, Myrtle Beach, USA; 4 School of Medicine, University of South Carolina, Columbia, USA; 5 Emergency Medicine, Orange Park Medical Center, Orange Park, USA; 6 Pediatrics, Memorial Health University Medical Center, Savannah, USA

**Keywords:** healthcare worker colonization, healthcare workers, methicillin-resistant staphylococcus aureus, bacterial colonization, cumulative incidence

## Abstract

Background

Methicillin-resistant *Staphylococcus aureus* (MRSA) can colonize up to 14.5% of healthcare workers (HCWs). The colonization rate of HCWs or the hospital setting that contributes most to MRSA colonization is less clear. In this study, we studied new resident physicians (PGY-1), as a model for HCWs, to measure their colonization rate and hypothesized that the incidence of colonization would increase during their first year.

Methodology

We prospectively enrolled PGY-1 residents of multiple specialties at three academic medical centers. After obtaining informed consent, PGY-1 residents were tested for MRSA in June 2019 before starting any clinical rotations and then retested every three to four months thereafter. The coronavirus disease 2019 pandemic forced us to end the study early. If MRSA-positive, residents were treated with 2% mupirocin and retested for a cure. For comparison, upper-level residents (PGY-2-5) were also enrolled to obtain a baseline prevalence of colonization.

Results

We enrolled 80 PGY-1 and 81 PGY-2-5 residents in the study. The baseline prevalence of MRSA colonization was 4.94% (4/81) in PGY-2-5 residents and 2.50% (2/80) for new PGY-1 residents; however, this was not statistically significant (p = 0.68). The cumulative yearly incidence of developing MRSA colonization in PGY-1 residents was 4.51%. MRSA colonization was successfully treated in 75% of cases.

Conclusions

PGY-1 residents had a lower MRSA colonization rate compared to PGY-2-5 residents, although this was not statistically significant. PGY-1 residents had a small incidence of developing MRSA colonization while working in the hospital. Further research is needed to determine if this is clinically relevant to HCWs or their patients.

## Introduction

Methicillin-resistant *Staphylococcus aureus* (MRSA) is a virulent strain of *S. aureus* that is resistant to β-lactam antibiotics and other antibiotics [1]. Together *S. aureus* and MRSA cause an enormous disease burden, including skin and soft tissue infections, postsurgical infections, pneumonia, bloodstream infections, sepsis, and endocarditis [2]. In 2005, 53% of *S. aureus* strains in the United States were MRSA [3]; however, this trend has been decreasing because of prevention strategies such as universal MRSA screening in patients, contact precautions, and hand hygiene initiatives [4]. Humans can be colonized with MRSA in various places (nares, axilla, vagina, pharynx, or skin) [5,6]. Colonization refers to the presence of bacteria that does not cause a detectable host immune response, cellular damage, or clinical signs and symptoms of infection. Humans colonized with MRSA are at an increased risk of developing a significant disease or subsequent infection [1,7-9]. Healthcare workers (HCWs) are frequently exposed to patients with MRSA in the hospital and have an increased rate of colonization, and the estimated rate of colonization varies from 0.2% to 14.5% among HCWs [10-13]. This large variance in prevalence tends to be higher in warmer climates [13-15] and acute care settings such as the Emergency Department [16]. A meta-analysis of HCWs in the United States and Europe showed the pooled prevalence rate of 31 different sites to be 4.4% [11]. The lowest prevalence occurred in cold climates or communities without known risk factors, where it was as low as 0.2% [12]. The concern with HCWs colonized with MRSA is that they may inadvertently pass it on to their patients; however, the frequency of this mode of transmission is not known. In addition, hospitals may have a responsibility to prevent employees from becoming colonized with MRSA as occupational exposure. This is akin to a common hospital employment requirement of annual influenza vaccines to prevent accidental transmission of influenza to HCWs or their families.

Newly graduated (postgraduate year or PGY-1) resident physicians are a unique group to study the rate of MRSA colonization among HCWs. First, as a group, they begin working in the hospital at the same time, July 1st, making it easy to capture a baseline rate of MRSA colonization before their first day of employment. Second, residents are a heterogeneous group and are exposed to a wide variety of hospital patients. At our medical centers, there are many residencies including Emergency Medicine, Internal Medicine, and General Surgery which enables good exposure to a wide variety of patient populations. Although residents have some clinic responsibilities, they primarily work in the hospital setting, throughout different wards, operating theaters, and during various shifts including nights and weekends. Lastly, PGY-1 residents are typically young (<30 years of age) and healthy, setting them up to be good vectors for new asymptomatic MRSA colonization.

We expected new residents’ baseline MRSA colonization rate to be between 1% and 3%, which is higher than the overall documented community rate (0.2%) but lower than the rate among HCWs documented in previous studies (4.3-4.6%) [10,11]. Our estimate was based on the assumption that prior to residency, the majority of residents recently completed medical school rotations and were likely exposed to MRSA during that time. As our facilities are in the warm, southern US climates and contain an active acute care setting (Emergency Department), factors responsible for higher MRSA colonization, we hypothesize that the cumulative incidence of MRSA colonization in residents after one year of employment will be higher than prior reports (4.3-4.6%). Secondarily, we tested cure rates for the standard treatment of MRSA colonization (2% mupirocin) in our study population and attempted to identify which type of resident physician was most at risk for MRSA colonization.

## Materials and methods

We conducted a prospective analysis of MRSA colonization among resident physicians of multiple specialties at three different academic medical centers (Sites 1-3) in the southeastern United States. Resident physicians employed as physicians for greater than one year (PGY-2-5) were considered to be our control population as they had been exposed to our specific hospital settings for more than one year. PGY-1 residents were considered to be our case population because they were new to our medical centers and had limited clinical exposure over the prior year. To be eligible, PGY-1 residents needed to have graduated from their medical schools in the immediate past term. Whereas PGY-2-5 residents included all upper-level residents including those who may have completed a prior transitional year residency or transferred from another residency program. Both groups were enrolled with an informed consent protocol approved by the institutional review board at our affiliated medical school (#2017-004). PGY-1 residents were approached at their new resident orientation at the beginning of July 2019 before starting any clinical duties in the healthcare setting. Combining all three medical centers, 142 PGY-1 resident physicians were approached during their orientation. PGY-2-5 residents (out of 226 total prior employed) were voluntarily recruited at their departmental didactic seminars in July 2019 and were provided instructions regarding where to go after didactics and how to enroll in the study. Both groups were provided the means to enroll privately and securely without the knowledge or undue influence of their program directors. The demographic information that we collected included the resident’s age, gender, program type, postgraduation year, and history of recent antibiotic use or surgeries in the prior 28 days.

To obtain MRSA colonization data, the investigators collected nasal swab samples from both nares. This was done by inserting a soft cotton swab into both nares for five seconds with a complete rotation of the swab. The samples were labeled with a unique subject number, kept at room temperature (25 ± 2°C) or lower, and analyzed within 48 hours of collection using a BD Max (Becton Dickinson, Franklin Lakes, NJ) using polymerase chain reaction (PCR) techniques. The BD Max operation manual allowed up to 48 hours from collection to analysis at room temperature and 120 hours with chilled storage. All samples were analyzed on the same machine at Site 1. Samples from Sites 2 and 3 were overnight mailed to Site 1 with chill packs and analyzed the next day. Any samples arriving at Site 1 more than 48 hours from the collection were discarded. The laboratory technician performing the PCR analysis was blinded to all relevant information except for the subject number and MRSA results and recorded the results on a spreadsheet. Other body areas were considered for MRSA sampling, but the BD Max analyzer is only approved by the manufacturer for nasal swabs.

Residents with positive MRSA results were notified by their local investigator and provided standard treatment with 2% mupirocin ointment twice a day for five days, and were then retested for cure after one week. The MRSA testing process for PGY-1 residents was repeated after three, six, and nine months of employment. For comparison, PGY-2-5 resident physicians were enrolled in the study during July 2019 to obtain a baseline prevalence of MRSA colonization in this population. The PGY-2-5 residents were similarly tested, treated with 2% mupirocin if indicated, and retested for cure after one week. PGY-2-5 residents were tested and treated only in July 2019, and not at the subsequent three, six, or nine months.

Statistical analysis

All sample size, power analysis, and statistics were calculated using Statistica software (TIBCO, Inc., Palo Alto, CA). Minimal sample size power analysis was performed prior to the initiation of the study. The minimum number of PGY-2-5 participants needed to verify an expected 4-5% prevalence of MRSA colonization, with a 5% margin of error, was 42-48 residents. Maximum PGY-1 enrollment (142 residents) provided 11-48% power to detect a difference in baseline prevalence versus PGY-2-5 residents with 95% confidence. To calculate MRSA colonization prevalence, we divided the number of MRSA-positive cases by the total number of subjects tested. To calculate the treatment cure rate, we divided the number of MRSA-positive cases treated and retested negative for cure by the total number of subjects treated. Lastly, to calculate the cumulative incidence of MRSA colonization, we divided the number of new PGY-1 MRSA colonizations identified over the entire study period by the total number tested. To be counted as a new MRSA infection, the resident had to test positive during the subsequent sample times (three, six, or nine months) as positive tests on enrollment were not counted as new. Colonization prevalence and cure rates were compared across time periods using a mixed-effects multinomial logistic regression model with random effects to account for repeated measures of individual residents. The baseline prevalence of MRSA among PGY-1 residents was compared to PGY-2-5 residents using Fisher’s exact tests. Statistical significance was predetermined as p < 0.05.

## Results

In July 2019, we enrolled 80 PGY-1 and 81 PGY-2-5 resident physicians into this study (subject characteristics are shown in Table 1).

**Table 1:** Subject characteristics. PGY: postgraduate year

Cohort	n	Age (years, mean)	Males
Total PGY-1	80	29.1	56%
Subgroups			
Anesthesiology	4	29.3	75%
Emergency Medicine	23	29.5	65%
Family Medicine	10	31.8	40%
General Surgery	3	27.0	67%
Internal Medicine	21	28.4	57%
Obstetrics & Gynecology	1	26.0	0%
Pediatrics	8	29.5	38%
Transitional Year	10	27.8	60%
Total PGY-2-5	81	30.8	52%
Subgroups			
Anesthesiology	1	29.0	100%
Cardiology	1	34.0	0%
Emergency Medicine	15	30.8	53%
Family Medicine	16	31.4	44%
General Surgery	14	31.0	50%
Internal Medicine	16	29.4	56%
Obstetrics & Gynecology	1	29.0	0%
Pediatrics	9	30.0	33%
Psychiatry	2	36.5	50%
Radiology	6	31.3	100%

The second quarter swabs were completed in October 2019 and the third quarter swabs in January 2020. The study was stopped after March 2020 as all face-to-face research was prohibited due to the coronavirus pandemic, and we were unable to complete the fourth quarter swabs (April 2020).

The baseline prevalence of MRSA colonization in our population (PGY-2-5 group) was 4.94% (4/81, Table 2), and for new PGY-1 residents before starting clinical shifts in June was 2.50% (2/80); however, this difference was not statistically significant (Fischer’s exact test, p = 0.68). Two PGY-1 subjects withdrew from the study during the first nine months. This left 76 PGY-1 residents to follow over time and calculate the cumulative incidence of developing MRSA colonization. The cumulative incidence of developing MRSA colonization for PGY-1 residents over the first seven months of employment (July to January) was 2.63% (2/76) or extrapolated to an annual rate of 4.51% per year (Fisher’s exact test, 95% confidence interval = 0.55-16.3%). The two incidences of new MRSA colonization occurred in Emergency Medicine and Internal Medicine residents, one case in each program. MRSA colonization was successfully treated with 2% mupirocin in 75% of all cases (6/8).

**Table 2:** Cases of MRSA colonization. MRSA: methicillin-resistant *Staphylococcus aureus*; PGY: postgraduate year; M: male; F: female

Baseline MRSA colonized control group (PGY-2-5 group)			
Program	Gender	Age (years)	PGY
Emergency Medicine	M	29	3
Family Medicine	F	27	2
Family Medicine	F	30	2
Internal Medicine	M	30	3
Baseline cases of MRSA colonization (PGY-1 group)			
Program	Gender	Age (years)	
Emergency Medicine	M	29	
Internal Medicine	M	28	
Incidences of new MRSA colonization (PGY-1 group)			
Program	Gender	Age (years)	Quarter
Internal Medicine	F	31	Second
Emergency Medicine	M	26	Third

## Discussion

Despite ending this study early, we were able to extrapolate some meaningful data about HCWs’ cumulative incidence of MRSA colonization. Multiple prior studies have characterized the point prevalence of MRSA colonization in HCWs in Europe, the United States, or worldwide and are quite variable based on the location, climate, clinical setting, and other factors. In 2008, Albrich and Harbarth [10] estimated the overall point prevalence to be approximately 4.6%. Later, a 2014 meta-analysis [11] of 31 studies regarding the point prevalence of MRSA colonization in HCWs found the pooled prevalence to be closer to 4.4%. In addition, they were able to show that HCW colonization rates were higher in the United States than in Europe (6.6% vs. 4.4%, p < 0.001). Furthermore, nurses seemed to have higher prevalence rates than other medical staff (odds ratio higher than 1.72); however, this was only true for Europe and there was no difference in the United States. The authors concluded this was because nurses, in general, have more frequent close encounters with MRSA-positive patients. Similarly, for students, as they progress through clinical rotations and have more frequent close encounters with patients, their point prevalence of MRSA colonization increases [17].

To our knowledge, our project is the first attempt to follow the same group of HCWs over time to determine their cumulative incidence (occupational risk) of developing MRSA colonization during their employment. Although our data is inconclusive, it suggests a small risk of HCWs developing MRSA colonization while working at medical centers. The number of new cases was too small to determine which residency program had the highest risk of developing MRSA colonization; however, our two cases occurred in residents training in Emergency Medicine and Internal Medicine. This may be consistent with prior studies that have indicated that MRSA colonization is more prevalent in HCWs who work in high-risk environments such as the emergency department [10].

In addition, our results showed that only 75% (6/8) of MRSA colonization cases were successfully treated with 2% mupirocin. Mupirocin is the accepted standard of care for colonization and works by inhibiting protein synthesis in *S. aureus* [18]. Mupirocin is also used in conjunction with chlorhexidine bathing, but we did follow that in our protocol. For our positive cases, we provided the ointment and a treatment diary. All eight subjects completed their treatment diaries and documented the application of 2% mupirocin twice a day for five consecutive days. Nevertheless, 25% of cases were not successfully eradicated. This is consistent with reports of mupirocin resistance developing among MRSA isolates [19].

HCWs colonized with MRSA pose significant risks to themselves, their families, and their patients. First and foremost, HCWs have approximately a 5% chance of developing a severe infection themselves [10,20,21]. No severe infections were reported to the investigators of this study. Intrafamilial spread of MRSA-infected HCWs has also been documented [22], thus introducing the risk of MRSA-related infections to one’s family. Lastly, MRSA-infected HCWs have been implicated as vectors for transmission of MRSA to their patients [10,23,24]. Therefore, colonization of MRSA in HCWs should be taken seriously, and we advocate for increased screening and preventative efforts among HCWs as an occupational disease.

Limitations

The coronavirus disease 2019 pandemic limited our ability to complete this project as planned. Because our institutional review board suspended all face-to-face research projects in early 2020, we could only get seven months of data instead of the planned 12 months. Consequently, we had to estimate our cumulative annual incidence based on only seven months of data. Furthermore, our study was limited by the size of enrollment. We had anticipated enrolling up to a maximum of 142 PGY-1 resident physicians from our three medical centers; however, only 80 (57.1%) volunteered to participate in the study. This was lower than our estimated enrollment and limited our power analysis. Despite this, our cumulative incidence of developing MRSA colonization was consistent with the prior reports of point prevalence. Lastly, our three medical centers are all in the southeastern United States, which limits the applicability of our results to other cooler or dryer regions.

## Conclusions

Resident physicians at our three medical centers in the southeastern United States had a baseline prevalence of MRSA colonization (4.94%) similar to previously reported values. The cumulative incidence of developing MRSA colonization was 4.51% per year. Mupirocin (2%) was moderately successful at eliminating MRSA colonization. This study adds to the available evidence that HCWs working in an academic medical center have a small risk of developing MRSA colonization. Further research is needed to determine if this leads to any illness among HCWs or leads to transmission and illness to their respective patients.
